# Effect of In‐Season Plyometric Training and Biological Maturation on Development of Slow and Fast Stretch‐Shortening Cycle Function in Youth Female Soccer Players

**DOI:** 10.1002/ejsc.70053

**Published:** 2025-10-08

**Authors:** Lee D. McGarrigal, Christopher I. Morse, David T. Sims, Georgina K. Stebbings

**Affiliations:** ^1^ Department of Sport and Exercise Sciences Manchester Metropolitan Institute of Sport Manchester Metropolitan University Manchester UK; ^2^ The Football Association Burton Upon Trent UK

**Keywords:** girls, leg stiffness, maturation, RSI, sprinting

## Abstract

The aim of this study was to determine slow (> 250 ms) and fast (< 250 ms) stretch‐shortening cycle (SSC) function in youth female soccer players at different stages of maturity and observe the effect of supplementing 8‐week soccer training with a low‐frequency (once‐per‐week) plyometric training (PT) programme on SSC function in this population. The main findings were that soccer plus PT resulted in significant improvements in slow (countermovement jump height) and fast (reactive strength index, leg stiffness and linear sprinting) SSC function in youth female soccer players, regardless of maturity (*p* ≤ 0.001), with two exceptions in the pre‐PHV group (*p* ≥ 0.281). In comparison, only two markers of fast SSC function improved following 8‐week soccer training without PT: one in the mid‐PHV group (*p* ≤ 0.05) and one marker in the post‐PHV group (*p* ≤ 0.05). This study is the first to demonstrate that soccer training supplemented with low‐frequency PT is more effective at improving slow and fast SSC function in youth female soccer players, regardless of maturity status, than soccer training alone. This information is useful for strength and conditioning practitioners working with youth female soccer players to inform future warm‐up and/or training programmes with this population that might improve playing performance and mitigate the risk of anterior cruciate ligament (ACL) injury in a population more susceptible to such injuries than boys.

## Highlights

- Plyometric jump training is an effective intervention to improve the physical fitness and SSC of youth female soccer players, including countermovement jump height, reactive strength index, leg stiffness and linear sprint performance.
- Plyometric jump training is similarly effective in pre‐, mid‐ and post‐PHV youth female soccer players in most SSC outcomes, except for 5 maximal hopping height and reactive strength index (in favour of mid‐ and post‐PHV).
- 8‐week, once a week plyometric training elicited a greater training effect than soccer training alone over the same duration and frequency.
- It is recommended that to increase the force needed to improve jump height, reactive strength index, leg stiffness and linear sprinting, strength and conditioning practitioners include a combination of vertical and horizontal plyometric exercises.

## 1 Introduction

Soccer is an intermittent sport where sprinting and jumping contribute to more than 50% of all goals scored (Faude et al. 2012). Thus, there is a need for strength and conditioning practitioners to test and develop the sprinting and jumping ability in soccer players (Taylor et al. 2022; Turner et al. 2013a, 2013b). In recent years, girls' participation in soccer has increased exponentially, with over 1 million girls aged 5–15 years now regularly playing soccer in England (Football Association 2024). Unfortunately, soccer poses the highest risk of anterior cruciate ligament (ACL) injury in adolescent girls compared to any other sport (Childers et al. 2025). Due to the sexual divergence in anatomy, hormonal profiles and movement biomechanics that occur during maturation (Childers et al. 2025; Mancino et al. 2024), youth female players also demonstrate a greater number of ACL injuries than youth male players during soccer (Beech et al. 2022; Bram et al. 2021; Childers et al. 2025; Robles‐Palazon et al. 2022; Sanchez‐Sanchez et al. 2025). One study also reported a higher prevalence of knee injuries in female soccer players aged 14–16 years compared to any other age group and boys of the same age (Solstad et al. 2025). Therefore, mitigating the risk of ACL injury is of great importance to strength and conditioning practitioners when working with youth female soccer players.

Plyometric training (PT) is a time‐efficient, safe, effective and popular method used by strength and conditioning practitioners to improve sprinting and jumping ability (Ramirez‐Campillo et al. 2020; Sanchez et al. 2022, 2020) and in mitigating ACL injury risk in female athletes (Baharuddin et al. 2020; Shams et al. 2021; Viswanathan et al. 2025). Research studies state that PT might be highly transferable into soccer game scenarios, due to a strong reliance on vertical and horizontal expressions of power when defending, shooting and attacking (Datson et al. 2014; Lees et al. 2010; Stolen et al. 2005). Although PT is used extensively in men's soccer, it is surprising that one meta‐analysis reported that only 3 of 90 PT studies in soccer included girls aged < 18 years (Ramirez‐Campillo et al. 2022), highlighting this as an important area for research.

Lower‐body plyometric exercises such as jumping, hopping and bounding are characterised by cyclic or spontaneous rapid eccentric contraction (deceleration or absorption of force) followed immediately by a concentric contraction of the same muscle (acceleration or propulsion of force), both of which are defined by the stretch‐shortening cycle (SSC) (Markovic and Mikulic 2010; Santos et al. 2024; Turner and Jeffreys 2010). The SSC has previously been categorised as slow (> 250 ms) or fast (< 250 ms) based on ground contact time (GCT) and angular displacement of the hips, knees and ankles (Schmidtbleicher 1992), with a countermovement jump (CMJ) defined as a slow SSC action (Van Hooren and Zolotarjova 2017), and hopping and sprinting defined as fast SSC actions (Komi and Nicol 2011; Talukdar et al. 2021). The proposed mechanical and neurophysiological mechanisms for the potentiation of force during SSC actions include reuse of stored elastic energy, active state development (cross bridges of actin and myosin), activation of the stretch reflex and the working range (Turner and Jeffreys 2010).

Two common measures used to assess fast SSC capabilities are the reactive strength index (RSI) and leg stiffness (Dallas et al. 2020; Lehnert et al. 2020; Lloyd et al. 2009). Both measures exhibit athletes' rebounding capabilities during fast GCT (< 250 ms) based on fast and efficient use of the SSC (Lloyd et al. 2009). These measures are associated with improved sprint speed and jump performance, alongside reduced risk of injury (Butler et al. 2003; Wilson and Flanagan 2008). Given the prevalence of ACL injuries in youth female soccer (Allen et al. 2016; Childers et al. 2025), the assessment of RSI and leg stiffness during repeated jump tests could provide practitioners a valuable insight into the physical condition of players. Despite this, there is currently a dearth of research investigating these factors in this population (Ramirez‐Campillo et al. 2022). Although RSI and leg stiffness have not been tested in in youth female soccer players, two studies have done so in youth female taekwondo athletes and gymnasts (Dallas et al. 2020) and post‐PHV volleyball players (Sylvester et al. 2024) with conflicting results. Interestingly, Dallas et al. (2020) found that while female gymnasts aged ∼9 years significantly increased RSI by 35%, RSI decreased by 28% in female taekwondo athletes aged ∼13 years (Dallas et al. 2020). In the same study, leg stiffness increased in the taekwondo athletes by 31% but remained unchanged in the gymnasts. In contrast, Sylvester et al. (2024) found that leg stiffness increased by 4.4%–9.5% (depending on hopping frequency), whereas RSI did not improve. These results imply that fast SSC function may be influenced by maturation, and is task and sport dependent. Assessing fast SSC measures alongside slower SSC tests such as the CMJ can collectively highlight to practitioners the developmental needs of their players to be addressed using PT.

Considering the prevalence of ACL injuries in youth female soccer, and that the RSI and leg stiffness can reduce the risk of injury in girls (Lehnert et al. 2020), and the reliability and validity of cost‐effective portable devices to determine such variables (Healy et al. 2016; Ruggiero et al. 2016), it would be pertinent for strength and conditioning practitioners working with youth female soccer players to measure RSI and leg stiffness. However, currently, there is a dearth of data for this population within the literature. To the best of the authors' knowledge, only two studies have aimed to develop the RSI and leg stiffness in girls following PT (Dallas et al. 2020; Sylvester et al. 2024). These studies, however, did not use youth female soccer players, which may have different SSC demands to the youth female gymnasts, taekwondo athletes and volleyball players used in the previous studies. Adding RSI and leg stiffness alongside CMJ and sprinting performance to a testing battery aimed at youth female soccer players may allow strength and conditioning practitioners to create a more complete SSC profile of their players (Taylor et al. 2022). Doing so may influence whether it is necessary to prioritise the development of the slow and/or fast SSC through subsequent PT sessions.

Undertaking PT once per week in youth female soccer players appears to have a positive effect (ES ≥ 1.34) on several SSC actions related to soccer, such as kicking distance, jump height and speed (Ozbar et al. 2014; Rubley et al. 2011). However, these studies did not measure the SSC function of female soccer players throughout maturation (as defined using pre‐, mid‐ and post‐peak height velocity [PHV]) (Emmonds et al. 2018), nor did they measure RSI or leg stiffness. As a symbiotic relationship between the development of the SSC following a training stimulus and the development due to growth and maturation might exist (Lloyd et al. 2015), it would be interesting to assess at which stage of maturation, if any, girls experience this “synergistic adaptation” that has previously been observed in boys (Asadi et al. 2017, 2018; Lloyd et al. 2015; Moran et al. 2017). Observing that one or more stages of maturity in girls are sensitive to PT may influence when strength and conditioning practitioners introduce this form of training to female soccer players (Myer et al. 2013). To the best of the author's knowledge, there are no studies that have examined the effectiveness of low‐frequency PT in the development of several slow and fast SSC tasks in youth female soccer players, with consideration given to maturity. Thus, the aim of this study was to determine CMJ height, RSI, leg stiffness and sprinting metrics in female soccer players during maturity (pre‐, mid, and post‐PHV) and identify if the implementation of a low‐frequency 8‐week PT training programme would improve the performance of these metrics in this population.

## 2 Methods

### 2.1 Study Design

**FIGURE 1:**
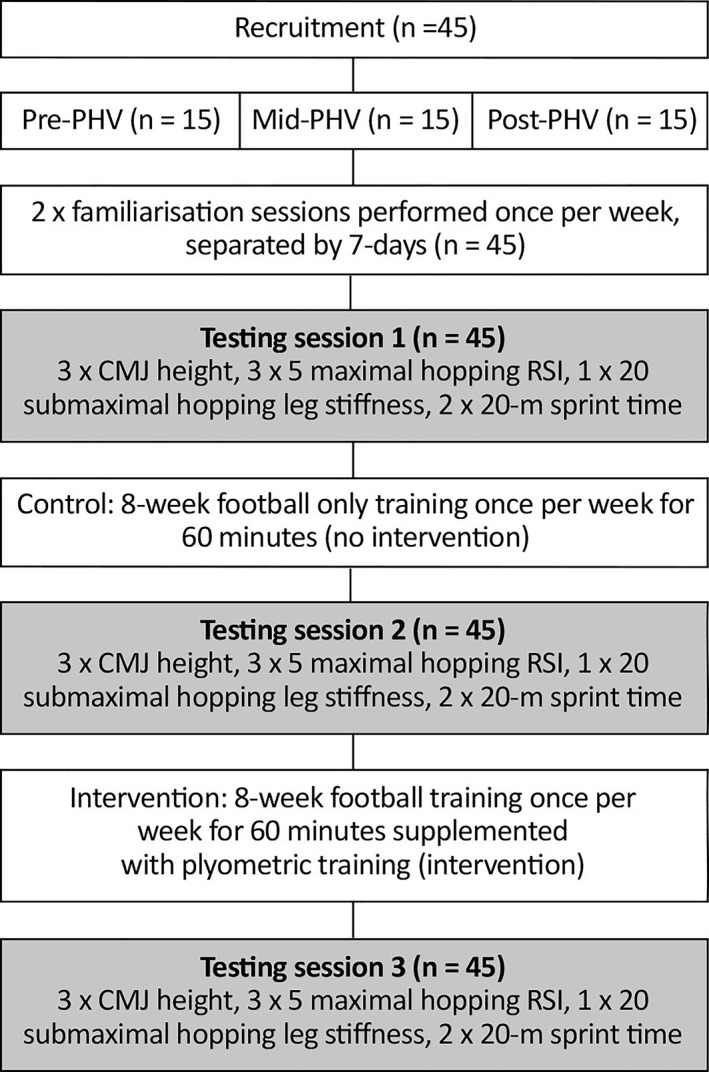
Summary of study design and experimental procedures.

This was a 16‐week crossover design which involved an 8‐week ‘control’ period (soccer‐only training) and an 8‐week ‘intervention’ period (Figure 1). Forty‐five recreational female soccer players were divided into three maturity groups based on maturity offset and age at PHV (Mirwald et al. 2002), with all participating in the control and subsequent intervention periods. Soccer sessions were of ∼60 min duration and were conducted once per week. The same duration and frequency of soccer was completed in the intervention period with 10–20 min PT once per week replacing the standard warm‐up of the 60 min soccer training, in addition to their normal physical education lessons (twice per week; 60 min per session) throughout the entire study. All tests were completed during the in‐season of the regular soccer calendar, with test 1 performed in week 1, test 2 performed in week 8 and test 3 completed in week 16, with the control period being between test 1 and test 2, and the intervention being between test 2 and test 3 (Figure 1).

### 2.2 Participants

Forty‐five youth female recreational soccer players from three squads within the same English soccer club (U10s, *n* = 15; U12s, *n* = 15; U16s, *n* = 15) with no prior experience of structured PT volunteered to take part in this study. Prior to the study, participants and parents were informed of the benefits and risks of PT, and only those that had signed parental consent took part. Throughout the study, participants were instructed to wear the same trainers and soccer kit (t‐shirt and shorts) during each testing point. The Ethics Committee of Manchester Metropolitan University (Manchester, UK) granted approval for this research study. All procedures complied with the latest Declaration of Helsinki (World Medical Association 2013).

### 2.3 Anthropometric Measurements

Standing and sitting height were measured to the nearest 0.1 cm with the use of a free‐standing stadiometer (Seca 213 stadiometer, Seca GmbH, Hamburg, Germany), with participants measured barefoot. Leg length was calculated by subtracting the sitting height from the standing height. Body mass was measured to the nearest 0.1 kg using calibrated digital scales (Seca 813, Seca GmbH, Hamburg, Germany). Body mass index (BMI) was calculated as body mass in kg divided by the square of height in metres. Anthropometric values are displayed in Table 1.

**TABLE 1:** Mean ± standard deviation (SD) of participants based on years from peak height velocity (PHV).

	Pre‐PHV (*n* = 15)	Mid‐PHV (*n* = 15)	Post‐PHV (*n* = 15)
Variable	**Mean ± SD**	**Mean ± SD**	**Mean ± SD**
Age (yrs.)	9.6 ± 0.4	11.8 ± 0.5	15.0 ± 1.1
Maturity offset (yrs.)	−2.1 ± 0.4	−0.2 ± 0.5	2.3 ± 0.8
Height (m)	1.40 ± 0.05	1.53 ± 0.04	1.64 ± 0.06
Body mass (kg)	31.9 ± 4.4	41.8 ± 5.7	57.3 ± 10.9
Body mass index (kg·m^2^)	16.2 ± 1.5	17.7 ± 2.5	21.3 ± 3.1
Leg length (m)	0.68 ± 0.03	0.75 ± 0.03	0.80 ± 0.05
Soccer training (yrs.)	2.4 ± 1.5	3.3 ± 2.0	5.8 ± 3.0

### 2.4 Age and Maturational Status

Chronological age (Table 1) was calculated as the difference between the date of birth and the date of first assessment (Emmonds et al. 2018). Maturity offset (Table 1) was estimated using a noninvasive, reliable regression equation (*R* ^2^ = 0.91, SEE = 0.50), where standing height, sitting height, leg length, body mass, age and the interaction between these variables are used to predict maturity offset and PHV (Mirwald et al. 2002). Players were then categorised as either pre‐ (< −1 year), mid‐ (−1 to +1 year) or post‐PHV (> + 1 year) based on the number of years from PHV (Emmonds et al. 2018).

### 2.5 Jump Testing

All jumping and hopping tests were in accordance with previous studies (Lloyd et al. 2009). Participants completed two familiarisation sessions to practice each test following a 5‐min standardised warm‐up (light jogging, jumping jacks and hopping). During each testing session, participants were instructed to perform each jumping and hopping task with their hands on their hips to avoid the interference of arm swing, which can elevate jump height (Cheng et al. 2008). Jumping and hopping flight time, jump height and GCT were measured using the Optojump Next system (Microgate, Bolzano, Italy). The Optojump Next consisted of two photoelectric parallel bars connected to a laptop via a USB cable, with one bar acting as a transmitter unit containing 96 light‐emitting diodes positioned 0.003 m above the ground, and the other bar acting as the receiver unit (Healy et al. 2016). Participants were instructed to stand between these parallel bars to perform each jump and hop. Flight time (ms) was recorded as the time between light transmission interruptions between the two bars, from which ground contact time (GCT) could then be calculated (Healy et al. 2016). The Optojump Next system was chosen due to the device's validity and reliability in measuring a countermovement jump (CMJ) (intraclass correlation coefficient [ICC] = 0.998; coefficient of variation [CV] = 2.2%), GCT (0.989), RSI (ICC = 0.985) and leg stiffness (ICC ≥ 0.800; CV = ≤ 6.8%) (Glatthorn et al. 2011; Healy et al. 2016; Ruggiero et al. 2016). To determine jump height, the flight time method (Equation 1) was used (Bosco et al. 1983)

(1) $$\text{Jump}\;\text{height}\;\left(\text{cm}\right)=\frac{\text{gravity}\;\left(9.81\;\text{ms}\right)\;x\;{\text{flight}\;\text{time}\;\left(\text{ms}\right)}^{2}}{8}$$

Unless otherwise stated, all tests were performed three times separated by 90 s to minimise the effect of fatigue (Meylan and Malatesta 2009), with the best measure used for further analysis (Lloyd et al. 2009). A maximal countermovement (CMJ) was used to assess leg power and slow (> 250 ms) SSC function (Lloyd et al. 2011; Turner and Jeffreys 2010). Prior to jumping, each participant adopted a foot stance shoulder‐width apart, with their hands on their hips (Glatthorn et al. 2011). On command, participants lowered themselves from an initial standing position to a self‐selected squat position, followed immediately by a rapid upward movement before take‐off (Lloyd et al. 2009). Participants were encouraged to perform the countermovement phase of the jump as quickly as possible, with the depth of the eccentric phase being self‐selected by the participant to maximise jump height (Cormack et al. 2008). The CMJ has been shown to be a valid and reliable test of vertical jump height in girls and boys aged 11–13 years (ICC = 0.97) (Dantas et al. 2020).

The 5 maximal (5max) hopping RSI involved participants performing five repeated maximal vertical hops (Lloyd et al. 2009), with participants instructed to ‘maximise jump height and minimise ground contact time’ (Dalleau et al. 2004). The first jump in each hop trial served as a CMJ and was discounted for analysis, with the remaining jump height and GCT averaged over four hops for subsequent RSI analysis (Lloyd et al. 2009), RSI was calculated using the equation popularised by Flanagan and Comyns (2008) outlined in Equation (2).

(2) $$\text{RSI}=\frac{\text{Jump}\;\text{height}\;\left(\text{mm}\right)}{\text{GCT}\;\left(\text{ms}\right)}$$

Leg stiffness was measured once during the 20 submaximal (20submax) hopping test at a frequency of 2.5 Hz (Lloyd et al. 2009), with hopping frequency maintained using a quartz metronome (SQ50 V; Seiko, Tokyo, Japan). During the 20submax hopping test, each participant was given one trial, and the ten consecutive hops where hopping frequency was closest to the designated metronome rate were used for further analysis (Lloyd et al. 2009). Leg stiffness was calculated using body mass (kg) flight time (ms) and GCT (ms) using the Dalleau et al. (2004) equation displayed in Equation (3):

(3) $$\text{Leg}\;\text{stiffness}=\frac{\text{Body}\;\text{mass}\times \pi \;\left(\text{flight}\;\text{time}+\text{GCT}\right)}{{\text{GCT}}^{2}\times ((\frac{\;\text{flight}\;\text{time}+\text{GCT}\;}{\pi })-(\frac{\text{GCT}\;}{4}))}$$

Leg stiffness was normalised for body mass and leg length to calculate relative leg stiffness (McMahon and Cheng 1990) as outlined in Equation (4).

(4) $$\text{Relative}\;\text{leg}\;\text{stiffness}=\frac{\text{Leg}\;\text{stiffness}\;x\;\text{leg}\;\text{length}\left(\text{mm}\right)}{\text{body}\;\text{mass}\;\left(\text{kg}\right)\;x\;\text{gravity}\;\left(9.81\right)}$$

To minimise lateral and horizontal displacement during performance, participants were instructed to jump and land on the same spot, land with legs fully extended (i.e. triple extension at the acetabulofemoral femorotibial and the talocrural joints) and to look forward at a fixed position to aid balance maintenance (Lloyd et al. 2009; Ramirez‐Campillo et al. 2015). Adherence to these instructions was carefully monitored by the lead researcher during all testing sessions. If a participant failed to comply with these instructions, the trial was deemed invalid and discarded. Following an invalid trial, the participant was reminded of the protocol and asked to perform the trial again following adequate rest (De Ste Croix et al. 2017; Lloyd et al. 2009).

### 2.6 Sprint Testing

Linear sprint time over 20‐m was measured using the Brower Timing II system photocell‐timing gates (Brower Timing Systems, Utah, USA). All photocells were mounted 50 cm above the ground on free‐standing tripods, as recommended by the Norwegian Olympic Federation (Shalfawi et al. 2012). Each pair of photocells was separated by 20 m on a G4 soccer pitch and participants were instructed to accelerate 0.5 m before the first timing gate and decelerate 10 m beyond the second timing gate, as identified by an additional marker (Loturco et al. 2013), with the lowest sprint time used for further analysis.

### 2.7 Plyometric Training

The duration of plyometric exercises in each session was ∼20 min, which were dependent on the planned session intensity and volume, and the sessions were separated by 7 days (Ozbar et al. 2014; Rubley et al. 2011). To maximise the vertical and horizontal force required to jump higher and run fast, plyometric exercises were performed in both the vertical and horizontal planes (Markovic and Mikulic 2010; Ramirez‐Campillo et al. 2015). Training volume was determined by the number of foot contacts made during each session (Jarvis et al. 2016) starting with 80 contacts in the first session, increasing to 190 contacts in the penultimate session, which tapered to 60 contacts in the final session to minimise fatigue (Ramirez‐Campillo et al. 2020) (Table 2). Plyometric drills lasted approximately 5–10 s, and 90 s rest was allowed after each set (Meylan and Malatesta 2009). All training sessions were supervised by the principal investigator using a trainer‐to‐participator ratio of 1:4, with particular attention paid to demonstration and execution (Ramirez‐Campillo et al. 2015). All training groups completed the same number of total jumps, on the same surface, and at the same time of day, with all rest intervals between each drill lasting 60 s (Ramirez‐Campillo et al. 2015). Aside from the formal PT, all participants completed their regular physical education classes.

**TABLE 2:** 8‐week (once per week) progressive plyometric training programme.

	Week 1	Week 2	Week 3	Week 4	Week 5	Week 6	Week 7	Week 8
Submaximal bilateral hopping	2 × 20	2 × 10	2 × 10	3 × 10	2 × 20	3 × 20	3 × 20	2 × 20
Maximal bilateral hopping	5 × 6	2 × 10	2 × 10	2 × 10	2 × 10	2 × 10	3 × 10	2 × 5
Countermovement jumps	10^a^	2 × 10	2 × 10	2 × 10	2 × 10	2 × 10	2 × 10	1 × 10
Unilateral vertical hopping		2 × 10	2 × 10	2 × 10	2 × 10	2 × 10	2 × 10	
Bilateral horizontal hops		2 × 10	2 × 10	2 × 10	2 × 10	2 × 10	2 × 10	
Unilateral horizontal hops			2 × 10	2 × 10	2 × 10	2 × 10	2 × 10	
Bounding					2 × 10	2 × 10	2 × 10	
Sprinting	20‐m × 2	10‐m × 3	15‐m × 2	20‐m × 2	30‐m × 2	50‐m × 2	50‐m × 3	20‐m × 2
Total foot contacts per leg^a^	80	100	120	130	160	180	190	60

^a^ Total foot contacts exclude foot contacts during sprints (Markovic et al. 2007).

### 2.8 Statistical Analysis

Data is presented as mean ± standard deviation (SD). A 3 × 3 (Group × Time) analysis of variance (ANOVA) was used as an omnibus test to observe the effect of PT over time (Test 1‐Test 3) and between groups (pre‐ mid‐ and post‐PHV). Where sphericity was not met, significance was assessed using Greenhouse‐Geisser correction. Normality and homogeneity of variance between groups were tested using the Shapiro‐Wilk and Levene's test. If significant between‐ or within‐group effects were observed, pairwise comparisons were conducted using the Bonferroni post‐hoc. If normal distribution assumptions were not met following ANOVA, Wilcoxon and Mann‐Whitney‐U tests were used for between‐ and within‐group comparisons, respectively, both with a Bonferroni correction of *α* = 0.05/2 used for the Wilcoxon test and a Bonferroni correction of *α* = 0.05/3 used for the Mann–Whitney‐U test. The effect size (ES) was determined using the modified Cohen's d scale, with ≤ 0.2 classified as trivial, 0.2–0.6 small, 0.6–1.2 moderate, 1.2–2.0 large, 2.0–4.0 very large, and ≥ 4.0 extremely large (Hopkins 2002). Statistical significance was set at *p* < 0.05. The SPSS version 27 (IBM Corporation, New York, USA) was used for further analysis.

## 3 Results

Table 3 demonstrates the between‐group and within‐group differences for the control period (Test 1 to Test 2) and following the plyometric intervention (Test 2 to Test 3).

**TABLE 3:** Mean ± SD for all dependent variables for each maturity group per test, *p*‐values between‐group differences, % change difference prior to soccer training (Test 1—Week 1), following soccer training (Test 2—Week 8), and before PT (Test 2—Week 8) and following PT (Test 3—Week 16), *p*‐values within‐group, and effect size (ES) using Cohen's d (95% CI) for Test 1–Test 2, and Test 2–Test 3 changes.

Variable	Test 1	Test 2	Test 3	Δ%		Δ%	
	Week 0	Week 8	Week 16	Test 1–Test 2	Test 1–Test 2	Test 2–Test 3	Test 2–Test 3
	Mean ± SD	Mean ± SD	Mean ± SD	%	ES (95% CI)	%	ES (95% CI)
CMJ (cm)							
Pre‐PHV	18.2 ± 2.1	18.2 ± 2.1	21.9 ± 2.1	0.2	0.01 (−0.74–0.76)	**20.3** ^j^	1.78 (1.03–2.53)
Mid‐PHV	**25.0** ± **5.3** ^b^	**25.5** ± **5.0** ^b^	**29.1** ± **4.6** ^b^	1.8	0.09 (−0.66–0.84)	**14.1** ^j^	0.75 (0.01–1.50)
Post‐PHV	**25.5** ± **4.0** ^g^	**25.5** ± **4.1** ^g^	**29.1** ± **4.2** ^g^	−0.3	0.02 (−0.73–0.76)	**14.4** ^j^	0.90 (0.15–1.64)
5max jump height (cm)							
Pre‐PHV	16.9 ± 3.2	17.4 ± 2.7	18.3 ± 3.8	3.3	0.19 (−0.56–0.93)	4.6	0.24 (−50–0.99)
Mid‐PHV	20.1 ± 5.0	21.0 ± 5.0	**23.5** ± **5.4** ^a^	4.5	0.18 (−0.57–0.93)	**12.1** ^j^	0.48 (−0.26–1.23)
Post‐PHV	**22.9** ± **4.5** ^g^	**23.7** ± **4.3** ^g^	**25.2** ± **3.4** ^g^	3.6	0.19 (−0.56–0.93)	**6.2** ^h^	0.37 (−0.38–1.12)
5max GCT (ms)							
Pre‐PHV	226.5 ± 32.6	232.3 ± 29.7	206.9 ± 42.3	2.6	0.19 (−0.56–0.94)	**−11.0** ^i^	0.70 (−0.05–1.44)
Mid‐PHV	**271.1** ± **57.9** ^a^	256.3 ± 49.3	215.5 ± 29.0	**−5.4** ^h^	0.27 (−0.47–1.02)	**−15.9** ^j^	1.01 (0.26–1.76)
Post‐PHV	239.5 ± 30.2	230.8 ± 27.9	208.2 ± 25.0	−3.7	0.30 (−0.45–1.05)	**−9.8** ^i^	0.85 (0.11–1.60)
RSI (mm/ms)							
Pre‐PHV	0.76 ± 0.16	0.77 ± 0.16	0.85 ± 0.32	0.9	0.04 (−0.70–0.79)	10.7	0.33 (−0.42–1.07)
Mid‐PHV	0.80 ± 0.38	0.87 ± 0.35	1.16 ± 0.44	**7.7** ^h^	0.17 (−0.58–0.92)	**34.5** ^j^	0.75 (0.00–1.50)
Post‐PHV	0.98 ± 0.27	**1.04** ± **0.25** ^e^	**1.23** ± **0.24** ^e^	**6.9** ^h^	0.26 (−0.49–1.01)	**17.7** ^j^	0.75 (0.01–1.50)
20submax GCT (ms)							
Pre‐PHV	220.5 ± 28.6	225.7 ± 28.1	194.3 ± 19.8	2.4	0.18 (−0.56–0.93)	**−13.9** ^j^	1.29 (0.54–2.04)
Mid‐PHV	242.2 ± 49.7	233.7 ± 44.9	197.0 ± 29.0	**−3.5** ^h^	0.18 (−0.57–0.93)	**−15.7** ^j^	0.97 (0.22–1.72)
Post‐PHV	213.5 ± 25.5	210.1 ± 21.5	187.0 ± 20.1	−**1.6** ^h^	0.14 (−0.60–0.89)	**−11.0** ^j^	1.11 (0.36–1.86)
20submax flight time (ms)							
Pre‐PHV	282.8 ± 46.2	293.1 ± 69.6	293.4 ± 64.63	3.7	0.18 (−0.57–0.92)	0.1	0.00 (−0.74–0.75)
Mid‐PHV	324.7 ± 73.2	332.1 ± 87.6	327.3 ± 61.7	2.3	0.09 (−0.66–0.84)	−1.4	0.06 (−0.69–0.81)
Post‐PHV	317.1 ± 70.4	335.9 ± 89.6	316.6 ± 80.4	5.9	0.23 (−0.52–0.98)	−5.8	0.23 (−0.52–0.98)
Absolute leg stiffness (kN)							
Pre‐PHV	10.17 ± 2.40	9.89 ± 2.42	12.65 ± 2.55	−2.8	0.12 (−0.63–0.87)	**27.1** ^j^	1.11 (0.36–1.86)
Mid‐PHV	11.64 ± 4.17	12.26 ± 3.84	16.04 ± 4.13	5.3	0.15 (−0.59–0.90)	**30.9** ^j^	0.95 (0.20–1.70)
Post‐PHV	**18.89** ± **4.79** ^d^ ^,^ ^g^	**18.98** ± **4.70** ^d^ ^,^ ^g^	**23.63** ± **6.10** ^d^ ^,^ ^g^	0.5	0.02 (−0.73–0.77)	**24.5** ^j^	0.85 (0.11–1.60)
Relative leg stiffness (kN·kg^−1^·m^−1^)							
Pre‐PHV	22.42 ± 4.67	21.66 ± 4.14	27.61 ± 3.84	−3.4	0.17 (−0.57 – 0.92)	**27.5** ^j^	1.49 (0.72–2.24)
Mid‐PHV	21.65 ± 8.00	22.67 ± 7.22	29.54 ± 7.33	4.8	0.14 (−0.61–0.88)	**30.3** ^j^	0.94 (0.20–1.69)
Post‐PHV	26.79 ± 6.10	**27.15** ± **5.33** ^e^	**33.56** ± **6.74** ^e^	1.3	0.06 (−0.69–0.81)	**23.6** ^j^	1.06 (0.31–1.80)
20‐m sprint time (s)							
Pre‐PHV	3.67 ± 0.17	3.66 ± 0.15	3.56 ± 0.11	−0.6	0.13 (−0.62–0.88)	**−2.8** ^h^	0.78 (0.03–1.53)
Mid‐PHV	**3.46** ± **0.34** ^a^	**3.45** ± **0.30** ^a^	**3.36** ± **0.31** ^a^	−0.3	0.04 (−0.71–0.78)	**−2.5** ^j^	0.29 (−0.46–1.03)
Post‐PHV	**3.17** ± **0.18** ^c^ ^,^ ^g^	**3.14** ± **0.18** ^d^ ^,^ ^g^	**3.08** ± **0.17** ^c^ ^,^ ^g^	−1.0	0.18 (−0.57–0.93)	**−2.0** ^j^	0.37 (−0.38–1.11)

Abbreviations: 20submax, 20 submaximal hops; 5max, 5 maximal hops; CMJ, countermovement jump; GCT, ground contact time; RSI, reactive strength index; T1, pre‐soccer‐only training1; T2, post‐soccer‐only training and pre‐PT; T3, post soccer and plyometric training.

^a^ *p* ≤ 0.05 between Pre‐ and Mid‐PHV groups (Bold).

^b^ *p* ≤ 0.001 between Pre‐ and Mid‐PHV groups (Bold).

^c^ *p* ≤ 0.01 between Mid‐ and Post‐PHV groups (Bold).

^d^ *p* ≤ 0.001 between Mid‐ and Post‐PHV groups (Bold).

^e^ *p* ≤ 0.05 between Pre‐ and Post‐PHV groups (Bold).

^f^ *p* ≤ 0.01 between Pre‐ and Post‐PHV groups (Bold).

^g^ *p* ≤ 0.001 between Pre‐ and Post‐PHV groups (Bold).

^h^ *p* ≤ 0.05 within‐group changes (Bold).

^i^ *p* ≤ 0.01 within‐group changes (Bold).

^j^ *p* ≤ 0.001 within‐group changes (Bold).

## 4 Between‐Group

There were significant between‐group differences in the CMJ, with the mid‐ and post‐groups generating significantly greater jump height than the pre‐PHV group in Tests 1–3 (*p* ≤ 0.001; Table 3), with no differences between the mid‐ and post‐PHV groups at any time point (Table 3). The post‐PHV group also generated greater 5max jump height than the pre‐PHV group in Tests 1–3 (*p* ≤ 0.001; Table 3), as did the mid‐ and post‐PHV groups in 5max jump height in Test 3 only (*p* ≤ 0.05; Table 3). The mid‐PHV group generated significantly greater 5max GCT than the pre‐PHV in Test 1 (*p* ≤ 0.05; Table 3), whereas the post‐PHV group recorded significantly greater RSI and relative leg stiffness than the pre‐PHV in Test 2 and 3 (*p* ≥ 0.05; Table 3). The post‐PHV group also recorded greater absolute leg stiffness than the pre‐ and mid‐PHV groups (*p* ≤ 0.001; Table 3) and significantly lower sprint time than both the pre‐ and mid‐PHV groups at each time point (*p* ≤ 0.001 and *p* ≤ 0.05, respectively; Table 3). The mid‐PHV group also recorded significantly lower sprint times than the pre‐PHV group in all three tests (*p* ≤ 0.05; Table 3). There were no between‐group differences in 20submax GCT or flight time (Table 3).

## 5 Within‐Group

The mid‐PHV group demonstrated significant improvements in 5max GCT, RSI and 20submax GCT between Test 1 and 2 (*p* ≤ 0.05; ES ≥ 0.17; Table 3), as did the post‐PHV group in RSI and 20submax GCT (*p* ≤ 0.05; ES ≥ 0.14; Table 3). Following PT, all groups generated significant improvements in CMJ height, 5max GCT, 20submax GCT, absolute and relative leg stiffness (*p* ≤ 0.001; ES ≥ 0.02; Table 3), as well as 20‐m sprint time (*p* ≤ 0.05; ES = 0.04; Table 3), with only the mid‐ and post‐PHV groups significantly improving 5max jump height and RSI following the PT intervention (*p* ≤ 0.05; ES ≥ 0.17; Table 3).

## 6 Discussion

To the best of the authors' knowledge, this was the first study to examine the effect of low‐frequency PT on both slow and fast SSC function in youth female soccer players, with consideration given to maturity offset. The main findings of this study were that, compared to soccer training alone, a supplementary PT intervention over 8 weeks significantly improved all measured key performance variables, except for 20submax GCT and flight times in all groups, and 5max jump height, RSI and 20submax GCT in the pre‐PHV group. Significant improvements in CMJ height, RSI, leg stiffness and sprinting (*p* ≤ 0.001) in all maturity groups may have practical relevance to youth female soccer players' on‐field performance, alongside physical development and injury prevention (Butler et al. 2003; Emmonds et al. 2018). Considering most goals scored in soccer involve sprinting or jumping (Faude et al. 2012) and that increase in RSI and leg stiffness can reduce ACL injuries in youth female athletes (Lehnert et al. 2020), it is recommended that strength and conditioning practitioners should include PT in the training of female soccer players to improve slow and fast SSC function and reduce the indices of ACL injury in this population, regardless of maturation status.

Improving the athletic ability in pre‐, mid‐ and post‐PHV athletes concurs with one meta‐analysis that reported that children throughout maturation responded equally well in vertical jump height and sprinting following PT (Ramirez‐Campillo et al. 2023). Importantly, the current study demonstrated that PT induces improvements in both slow‐ and fast‐SSC related variables above what would be expected by growth and maturity alone, suggesting that the current cohort experienced synergistic adaptation (Lloyd et al. 2015). The fact that the mid‐ and post‐PHV groups increased all dependent variables, except 5max flight time following PT, but the pre‐PHV group did not (5max jump height and RSI), suggests that synergistic adaptation of the SSC is task‐ and maturity‐dependent, and that greater improvements following training can be expected in girls during and following PHV, due to an increase in power which occurs at the onset of the adolescent growth spurt (Beunen ansd Malina 1988).

The marked improvement by all maturity groups in CMJ, leg stiffness, relative leg stiffness and sprint performance following PT indicates the prescribed PT programme was effective, and that youth female soccer players can effectively improve the slow‐ and fast‐SSC when vertical and horizontal PT is integrated into their regular soccer training. These findings agree with much of the research studies demonstrating that PT is an effective modality of training to improve “explosivity”, such as CMJ (Hewett et al. 1996; Moran et al. 2018; Ozbar et al. 2014) and the limited studies in the RSI and leg stiffness capabilities amongst girls (Dallas et al. 2020; Sylvester et al. 2024). However, our findings demonstrate, for the first time, that low‐frequency PT is effective at developing RSI and leg stiffness in youth female soccer players. Given the positive relationship between greater RSI and leg stiffness in the reduction of ACL injuries (Brazier et al. 2014; Flanagan and Comyns 2008), it is recommended that coaches working within youth female soccer should use supplementary PT to develop these metrics, as doing so would allow coaches to make data‐driven decisions about their players' athleticism and ACL injury risk, which would inform future PT programme design (Emmonds et al. 2018; Lehnert et al. 2020).

As anticipated, there was a general trend that more mature participants outperformed their less mature counterparts (post‐PHV > mid‐PHV > pre‐PHV), particularly in CMJ height, leg stiffness and sprinting prior to and following PT (Table 3). These results might be linked to the more mature groups having ∼19 kg greater body mass, of which ∼14 kg is estimated as muscle mass, compared to the pre‐PHV group. Certainly, the estimated increase in muscle mass would contribute to greater concentric and eccentric force development, and hence greater CMJ, leg stiffness and sprint performances were observed in the mid‐ and post‐PHV groups compared to those of the pre‐PHV group. It is recommended that future research incorporates direct assessment of muscle mass, through dual‐energy X‐ray absorptiometry, as this is necessary to confirm these estimations. Based on the current findings, and that muscle mass increases prior to and during the adolescent growth spurt (Beunen and Malina 1988; Lloyd and Oliver 2012), practitioners should consider players' maturation status when attempting to benchmark their longitudinal SSC development and subsequent injury risk.

It has been stated that SSC is governed by neural regulation (Radnor et al. 2018), and that pre‐PHV athletes may be particularly sensitive to the high neural demands of PT (Lloyd et al. 2015), due in part to central nervous system maturation (Myer et al. 2013) and the heightened neural plasticity of this population (Lloyd et al. 2015; Ramirez‐Campillo et al. 2023). Improved jumping performance in all groups, however, could be attributed to factors such as increase in leg power, greater inter‐muscular coordination, enhanced neural drive to agonist muscles, changes in muscle size and architecture, changes in single fibre metrics as well as muscle–tendon mechanical stiffness, that can result in overall better utilisation of the slow SSC (Markovic and Mikulic 2010; Taube et al. 2012) and minimise the risk of injury in youth female soccer players (Chimera et al. 2004). Due to the short duration of the PT in the current study, however, physiological adaptations can most likely be attributed to neuromuscular improvements such as motor unit recruitment and firing rate (Markovic and Mikulic 2010; Sale 1988). Integrating assessments of neural function and skeletal muscle structure in future would be a useful addition to fully elucidate the contribution of these likely determinants in the observed improvements in jump performance following low‐frequency PT in maturing girls.

In the present study, RSI significantly increased in both the mid‐ (34.5%) and post‐PHV (17.7%) groups following PT, but not the pre‐group (10.7%). Such improvements by the mid‐PHV group are comparable with the 35% increase in RSI observed in a previous study involving female rhythmic gymnasts aged 8.4 years (Dallas et al. 2020). Our findings, however, are lower than the 53.5% improvement in RSI observed in female rhythmic gymnasts aged 10.8 years (Ng et al. 2023). This discrepancy is likely explained by the greater training volume used in Ng et al. (2023) compared to the current study. In addition, the elite‐level athletes of the Ng et al. (2023) study may have been more experienced with PT‐style training than the current cohort with no previous PT experience. Nevertheless, the significant improvements in RSI of the mid‐ and post‐PHV groups following PT in the present study may indicate these groups optimised muscle elasticity and neuromuscular control of working muscles more effectively than the pre‐PHV group post‐PT (Flanagan and Comyns 2008; Jarvis et al. 2021), and/or increased their tolerance to the eccentric loading of the musculotendinous unit (MTU) during maximal hopping to overcome high impact forces (Ng et al. 2023). Neuro‐mechanical adaptations, such as increased motor unit recruitment (Dallas et al. 2020), improved excitability of the neural receptors (Turner and Jeffreys 2010), a quicker rate of force development (RFD) and greater stretch–reflex contribution following PT (Davies et al. 2015; Matavulj et al. 2001) might explain these improvements in RSI of the mid‐ and post‐PHV groups. Indeed, considering the relevance of the RFD for youth soccer long‐term athletic development (Meylan et al. 2014), the observed improvement in RSI could enhance physical qualities related to on‐field soccer performance (Ramirez‐Campillo et al. 2018).

Compared to the pre‐PHV group, the mid‐ and post‐PHV groups may also have dampened the Golgi tendon organs, resulting in more spontaneous neuromuscular coordination, which is characterised by the nervous system generating faster muscle‐firing patterns and greater neural efficiency (Ng et al. 2023). As it is generally accepted that RSI is an important measure of SSC and rebound capability of youth athletes related to athletic ability (Flanagan and Comyns 2008; Jarvis et al. 2021; Ramirez‐Campillo et al. 2018) and injury prevention (Raschner et al. 2012; Toumi et al. 2006), finding that RSI did not increase in the pre‐PHV group suggests that younger girls should be exposed to greater PT volume, particularly as this population may be more sensitive to neuromuscular training including plyometric exercises (Myer et al. 2011; Ramirez‐Campillo et al. 2023). As the pre‐PHV group demonstrated significantly lower CMJ height and greater sprint time compared to the mid‐ and post‐PHV groups, it is possible that the pre‐PHV girls experienced more limited slow and fast SSC development due to a lower training age compared to the rest of the sample. Nevertheless, it is possible that all girls during maturation may benefit from increased PT volume, given the comparisons with other sports. Therefore, future researchers may wish to compare the changes in slow and fast SSC function in youth female soccer players using a variety of PT training volumes.

It has been demonstrated that one of the physiological adaptations following PT is increased musculotendinous stiffness (Cornu et al. 1997). In the current study, the training intervention led to a significant increase in absolute and relative leg stiffness in all maturity groups. Increases in leg stiffness are believed to occur due to minimal GCT, greater flight time, higher body mass or a combination of one or more of these factors (Farley et al. 1991; Korff et al. 2009; McMahon et al. 2012). Moreover, leg stiffness is governed in part by preactivation and short‐latency stretch reflexes (Hobara et al. 2007), with up to 97% of the variance in leg stiffness explained by the contribution of preactivation and the stretch–reflex response of lower limb extensor muscles (Oliver and Smith 2010). Observing an increase in leg stiffness, regardless of maturity offset, in the current study is pertinent considering that this metric, alongside RSI, is a key indicator of injury susceptibility in female sport, particularly in relation to ACL injury (Lehnert et al. 2020). We are confident therefore that by including even low‐frequency PT alongside soccer training, youth female soccer players will improve not only key performance indicators but also help prevent serious long‐term injury through the improvement of said variables.

Sprint time also improved in all maturity groups following PT, with the post‐PHV group again being the physically dominant group. Sprinting involves both slow (e.g., acceleration phase) and fast‐SSC (e.g., brief ground contacts) components of the SSC, and relies on the interaction of several other physiological and physical factors, including technique (stride length and frequency), lower body strength and power, musculotendinous stiffness, RFD and vertical force and horizontal force to increase forward propulsion (Lockie et al. 2015; Meylan et al. 2014; Suchomel et al. 2016; Washif and Kok 2022). In the current study, we observed improvements in CMJ height (a surrogate of concentric strength, leg power, and slow‐SSC), RSI (a measure of eccentric force absorption, transfer of energy and fast‐SSC), leg stiffness (a measure of hysteresis) (i.e., energy dissipation) and GCT (a measure of impulse) following PT; thus, it is unsurprising that sprint time decreased in all groups (Carr et al. 2015; Lockie et al. 2015; Meylan et al. 2014; Schmidtbleicher 1992; Washif and Kok 2022).

It was also important to incorporate both vertical and horizontal plyometric exercises, as horizontal PT causes greater adaptations in 0–10‐m sprint acceleration (Loturco et al. 2015), whereas vertical PT favours jump height (Markovic 2007) and sprinting speed from 10 to 20‐m (Loturco et al. 2015). In line with the principle of training specificity, the current study also included sprinting drills as part of the PT programme (Sale 1988; Markovic and Mikulic 2010). This is pertinent, given that sprinting is often seen as the purest form of PT and true measure of fast SSC function (Hansen and Kennelly 2017; Komi and Nicol 2011). Thus, it is recommended that, regardless of maturity, strength and conditioning practitioners should design PT programmes aimed at youth female soccer players, including sprinting drills as well as plyometric exercises in both the vertical and horizontal planes (Markovic 2007; Markovic et al. 2007; Markovic and Mikulic 2010; Ramirez‐Campillo et al. 2015; Kurt et al. 2023).

It should be acknowledged that this study had several limitations. For example, this study used PHV as a proxy for biological maturity. This method is commonly used in sporting contexts because it relies on standard anthropometric measurements that are easily and routinely collected by practitioners (Lloyd et al. 2014). Although more accurate methods, such as skeletal age assessment or clinical evaluation using Tanner stages, are considered the gold standard (Lloyd et al. 2014; Malina et al. 2004), they are less accessible. Skeletal age assessment requires portable X‐ray equipment and trained personnel to obtain and interpret wrist radiographs, whereas Tanner staging requires a qualified health professional to conduct potentially sensitive physical examinations (Lloyd et al. 2014). The latter, particularly the assessment of breast and pubic hair development, may cause discomfort or distress among adolescent female athletes (Moon and Davies 2023). Consequently, many studies opt for the less invasive PHV method (Davies et al. 2019; Malina et al. 2021; Sanchez et al. 2020; Sylvester et al. 2024; Talukdar et al. 2021) in favour of Tanner staging (Romero et al. 2019). Nevertheless, future research involving youth female soccer players may consider conducting assessments of skeletal age or Tanner staging, when feasible and ethically appropriate, with the support of qualified health professionals (Lloyd et al. 2014).

This study also used the Optojump Next device to measure flight‐time and GCT, from which jump height, RSI and leg stiffness were calculated. Although a laboratory‐based force platform could have provided a more comprehensive analysis of SSC function, including force‐power‐time curves, modified RSI, impact peak forces and spring‐like behaviour (McMahon et al. 2018; Moeskops et al. 2022; Pedley et al. 2021, 2020; Suchomel et al. 2015), the Optojump Next offers some practical advantages. It is more cost‐effective, portable and suitable for field testing, offering real‐time feedback and the flexibility to set up on any flat, sport‐specific surface, which enhances ecological validity (Garcia‐Lopez et al. 2013). Additionally, as the Optojump Next has demonstrated excellent reliability in the measurement of jump height, RSI and leg stiffness (Glatthorn et al. 2011; Healy et al. 2016; Ruggiero et al. 2016), it is a valuable tool for strength and conditioning coaches working with a large number of athletes in applied settings. Nonetheless, future studies may benefit from using a force platform to obtain a more detailed understanding of slow and fast SSC function in youth female athletes.

## 7 Conclusion

This is the first study to demonstrate that structured low‐frequency PT over 8 weeks, in addition to technical soccer training, improves CMJ height, leg stiffness, and linear sprint speed in recreational pre‐, mid‐, and post‐PHV female soccer players, regardless of maturity offset. Improvements in RSI were also observed but in more mature players only (Lloyd et al. 2012). These results support the theory that girls experience synergistic adaptation (Lloyd et al. 2015), suggesting that SSC function of youth female soccer players can be amplified by PT beyond the development of the SSC due to growth and maturation (Lloyd et al. 2015). Supplementing soccer training with PT is a time‐efficient, safe and effective form of training and, therefore, it is recommended that strength and conditioning coaches working with youth female soccer players incorporate plyometric exercises into practice sessions, especially in the competition season where less time is available for training (Ozbar et al. 2014). It would be useful for strength and conditioning coaches to monitor and develop RSI and leg stiffness during training in this population, given that improving these metrics may reduce the number of ACL injuries in a population susceptible to such knee damage. As this study was conducted in recreational youth female soccer players (with limited PT exposure), it remains unclear if similar results would be observed in elite players or those with prior experience of performing PT. Further work is needed to fully understand the effectiveness of PT in elite youth female soccer players, whether there is a minimal dose‐response to such training for youth females, and whether or not dose‐response changes vary with age and maturation.

## Conflicts of Interest

The authors declare no conflicts of interest.
